# The experiences and decision making of patients with incurable cancer and health literacy difficulties

**DOI:** 10.1371/journal.pone.0309104

**Published:** 2024-10-03

**Authors:** Chloe E. Holden, Richard Wagland, Amélie Harle, Sally Wheelwright

**Affiliations:** 1 Dorset Cancer Centre, University Hospitals Dorset NHS Foundation Trust, Poole, Dorset, United Kingdom; 2 Health Sciences, University of Southampton, Southampton, Hampshire, United Kingdom; 3 Sussex Health Outcomes Research & Education in Cancer (SHORE-C), Brighton & Sussex Medical School, University of Sussex, Brighton, East Sussex, United Kingdom; UT Austin: The University of Texas at Austin, UNITED STATES OF AMERICA

## Abstract

**Objective:**

Shared decision making is important when decisions are preference sensitive, as in incurable cancer. A prerequisite for shared decision making is health literacy, which is essential to facilitate good understanding of an individual’s current situation, the decision to be made, and the options available to them. This study sought to learn about the challenges for shared decision making faced by patients with incurable cancer and health literacy difficulties.

**Methods:**

Semi-structured telephone and video interviews were used to collect data on participants’ experiences, decision making, and challenges faced. Study procedures followed health literacy principles, with information offered in various formats to suit individuals’ preferences, the use of a verbal consent process, and flexibility in whether interviews were conducted over telephone or video call. Data were analysed using Framework Analysis (Ritchie et al. 2003), with initial verbatim transcription of interviews, iterative development of the analysis framework, indexing using Nvivo 12 software and summarising of the data before systematic categorisation and development of final themes.

**Results:**

Twenty participants (aged 31–80, of whom 13 male) with a variety of cancers (including breast, central nervous system, gastrointestinal, gynaecological, lung, head and neck, and urological) and experience of a range of treatments were interviewed. Seven themes were identified, including: supportive staff in an imperfect system, additional pressure from COVID-19, in the expert’s hands, treatment not so bad, emotional hurdles, accessing information to further understanding and wanting to be a good patient.

**Conclusion:**

In order to support patients with incurable cancer and health literacy difficulties to become involved in decisions about their care, we must address the emotional, social and informational challenges they face. Recommendations for achieving this include addressing peoples’ emotional needs, facilitating control over information, developing a partnership, involving others, and organisational changes.

## Introduction

Health literacy (HL) describes “the combination of personal competencies and situational resources needed for people to access, understand, appraise and use information and services to make decisions about health. It includes the capacity to communicate, assert and act upon these decisions” [1]. Three levels of HL have been described, from functional, which results in increased knowledge and compliance, through interactive HL, involving the development of personal skills and confidence, to critical HL, which leads to greater social and political action through increased empowerment [2]. Patients with lower HL tend to have greater difficulty understanding and processing cancer related information, poorer quality of life and a poorer experience of care [3]. HL is also a prerequisite for shared decision making (SDM) [4–6], which is where patients and clinicians work together, sharing information about options and preferences to agree on the best course of action for the individual [7]. SDM is important in palliative oncology, when patients may face difficult decisions such as whether to receive treatments aimed at controlling rather than curing the cancer, particularly when such treatments may offer marginal benefits at potentially significant risk, or focussing on managing symptoms as and when they occur.

Existing studies exploring HL and SDM in oncology have been quantitative evaluations [8–12], with mixed findings regarding an association between the two, perhaps in part due to the different HL and SDM measures used [3]. Little is known about the specific challenges faced by patients with incurable cancer who might be unable to access and understand information through conventional routes.

After undertaking an initial systematic review, which highlighted the association between health literacy difficulties and poorer outcomes in cancer care [3], the aim of this qualitative study sought to gain a deeper understanding of the challenges faced by patients with HL difficulties and incurable cancer. The objectives of the study, conducted during the COVID-19 pandemic, were therefore: 1) to understand the experiences and decision making of patients with lower HL receiving care for incurable cancer in the NHS; and 2) to identify the particular challenges faced by these patients.

## Methods

### Participants

Potential participants were identified by oncology teams in two NHS district general hospitals. Clinicians were provided with a list of ‘red flag’ behaviours and responses suggestive of possible difficulties with HL [13] to aid identification. Eligible patients were aged 18 years or over, had incurable solid or haematological cancer of any type and suspected HL difficulties, had capacity to provide informed consent and spoke English as a main language. Following identification, the clinicians made the initial approach, briefly introducing the study and obtaining permission from those interested for CH to make contact and provide further detail.

### Study procedure

Interested patients were telephoned by CH, a female medical oncology registrar (MBBCh) with nine years’ clinical experience and undertaking a PhD exploring SDM in palliative oncology. Participants were aware of CH’s medical background, but care was taken to ensure they had not met clinically. CH used a ‘layered approach’ [14] to giving information, initially providing a verbal explanation of the study, then offering further information in the form of a standard participant information sheet (PIS), single summary page, summary video (by email or posted video card), or any combination of these, which was sent to interested individuals according to patient preference. The full PIS was available on request at any time. Interviews were arranged at a time to suit the individual.

In light of restrictions imposed by the COVID-19 pandemic, telephone and video interviews were conducted and recorded through Microsoft Teams by CH, and verbal consent was recorded using teach-back to confirm understanding [15]. Interviews followed a topic guide (S1 File) framed around Nutbeam’s levels of health literacy [2], agreed by the research team and piloted with two PPI members. CH explored participants’ experiences, involvement in decision making, and challenges faced. Some participants chose to have relatives or other supporting persons present, however, as the study sought to learn from patients themselves, and only patients consented to take part, comments made by relatives or others present in some interviews were not included. Such comments were removed from the transcripts and excluded from analysis. Reflexive notes were made after the interviews and the research team met regularly to discuss progress.

Three HL screening questions were asked at the end of the interview [16]: 1. “How often do you have someone (like a family member, friend, hospital/clinic worker or caregiver) help you read hospital materials?”; 2. “How often do you have problems learning about your medical condition because of difficulty understanding written information?”; and 3. “How confident are you filling out forms by yourself?”. Any response of “some”, “most”, “all of the time” to questions 1 or 2, or “somewhat”, “a little bit”, “not at all” to question 3 was used to identify those experiencing difficulties. In line with the study’s ethical approval, the HL screening questions were only asked once consent had been obtained. The questions were asked after rather than before the interview, to allow development of rapport and to avoid creating unease or a test like environment which may have affected participants’ subsequent responses. Demographics (age and sex), date and type of cancer diagnosis, date of incurable disease, stage, treatments received, and comorbidities were obtained from medical records.

NHS Health Research Authority approval, including for obtaining recorded verbal rather than written consent, was obtained prior to research activities being undertaken (REC reference 20/PR/0478).

### Data analysis

Data were analysed using Framework Analysis [17, 18]. Interview recordings were transcribed verbatim by CH, allowing data familiarisation. CH and RW independently read the first six transcripts, noting initial thoughts and themes. They regularly discussed codes used in the initial framework, which was iteratively revised and agreed by the research team. The main framework themes were based on the research objectives, whilst subthemes included those introduced through interview questions and recurring themes in the data. Each transcript was indexed by CH, using NVivo 12 software. Thematic charts were created and the data within summarised, before further exploration of themes across cases. A table was drawn for each column of each matrix, identifying the different elements, then assigning categories. For example, all comments relating to emotions affecting participants’ ability to process information were collated and summarised, before identifying common elements such as the shock of diagnosis impairing ability to take in what was said, and being in a better position further down the line. These were grouped into categories, such as ‘difficult to process at beginning’ and ‘ready to take more in later’ which were brought together under overarching classifications, forming the final themes. Recruitment continued until no new information was being generated, suggesting data saturation [19].

Participants were not asked to provide feedback, but those alive after the final interview were offered a summary of preliminary findings.

## Findings

**Fig 1:**
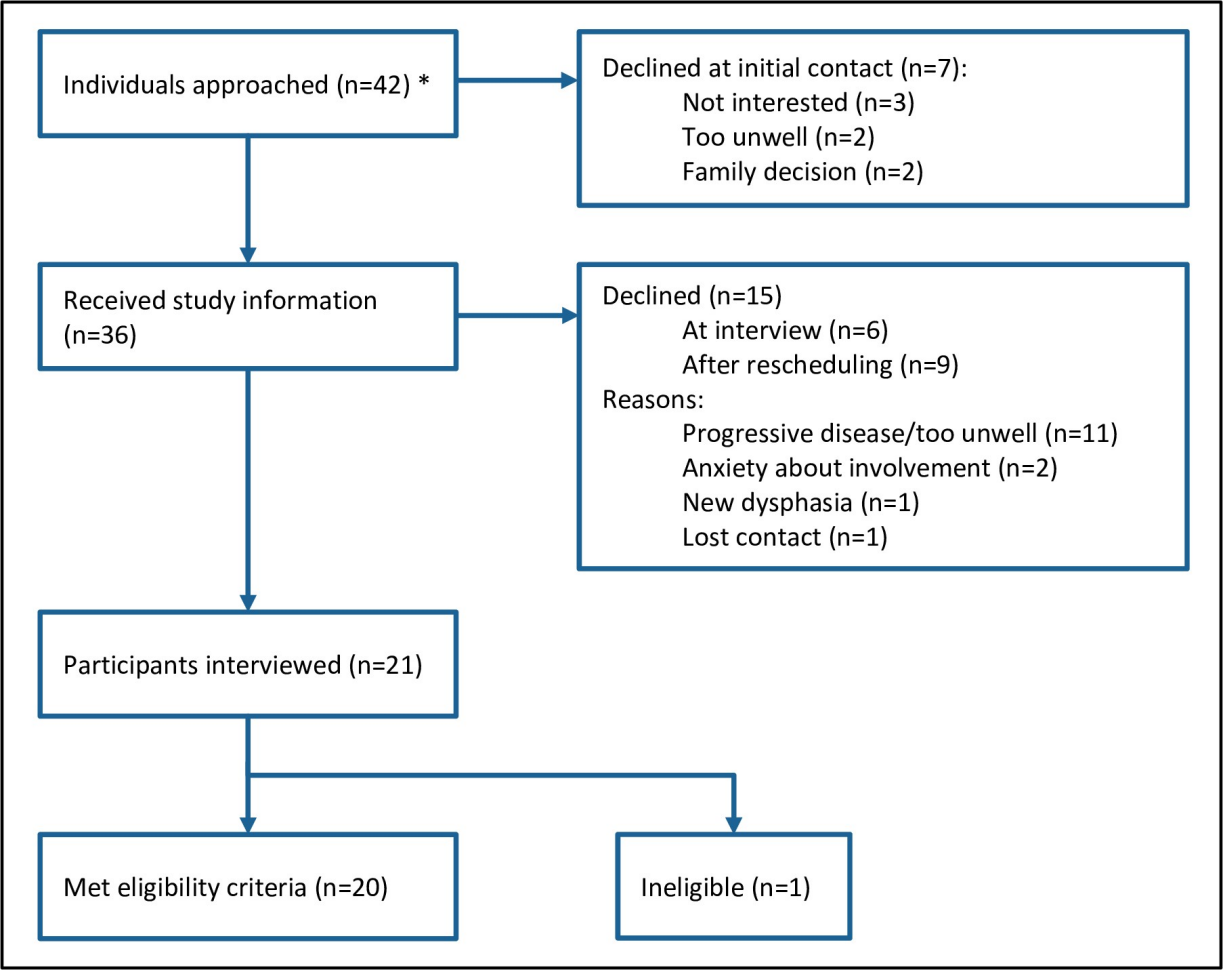
Participant recruitment and reasons for non-participation. * One potential participant was approached twice, therefore 43 total approaches were made. This individual initially declined after the first contact and subsequently received study information but later decided not to participate.

Forty-two potential participants were approached, and 21 interviews conducted between 21^st^ October 2020 – 29^th^ October 2021 (Fig 1). One potential participant was approached twice, making 43 total approaches. This individual initially declined after the first contact and subsequently received study information but later decided not to participate. One participant was identified after interview as receiving adjuvant treatment and did not meet eligibility criteria. Their responses were not analysed with the rest of the data as the nature of conversations and decisions made were different, but points relevant to their experience of the system were considered alongside the completed analysis and were found to be in support of the key themes identified from the remainder of the dataset.

Based on the screening questions, twelve participants met criteria for HL difficulties (HLD), and eight reported no HL difficulties (NHLD). The original protocol planned separate analyses for participants with and without HL difficulties based on the screening questions. Our preliminary analyses, however, identified contradictions between responses to the screening questions and interview responses, as well as other discrepancies discussed further in the Limitations, suggesting the screening questions did not discriminate between the two groups in this study. In light of this, and as all participants had been identified by healthcare professionals as appearing to have difficulties with HL, all data were analysed together.

### Participant characteristics

Nineteen participants were aged 51 years or over, and two thirds were male (**Table 1**). Participants had various cancer diagnoses, and most had recently received anti-cancer treatment.

**Table 1:** Participant characteristics.

Characteristic	Participants (n = 20)
**Age range**	
31–40	1
41–50	0
51–60	5
61–70	6
71–80	8
**Sex**	
Female	7
Male	13
**Primary tumour**	
Breast	1
Central nervous system	3
Upper gastrointestinal	2
Lower gastrointestinal	3
Ovarian	2
Lung	4
Head and neck	2
Prostate	2
Renal	1
Treatments received *	
Surgery	7
Chemotherapy	13
Radiotherapy	13
Immunotherapy	4
Hormone therapy	3
Targeted agents	3
**Anticancer treatment <3 months ago**	
Yes	16
No	4
**Health literacy according to Chew questions**	
Health literacy difficulties (HLD)	12
No health literacy difficulties (NHLD)	8

* Total greater than 20 as some participants received multiple treatment modalities

**Fig 2:**
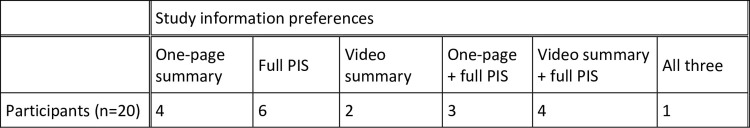
Study information preferences.

Participants’ preferences for study information differed (Fig 2). Most interviews were conducted by telephone (15/20), lasting a median of 52 minutes (range 33–109).

Seven themes were identified from the data. These are presented below according to the study objectives:

- The experiences and decision making of patients suspected of having HL difficulties whilst receiving care for incurable cancer in the NHS
   ⚪ Supportive staff in an imperfect system
   ⚪ Additional pressure from COVID-19
   ⚪ In the expert’s hands
   ⚪ Treatment not so bad
- Challenges faced by patients suspected of having HL difficulties whilst receiving care for incurable cancer
   ⚪ Emotional hurdles
   ⚪ Accessing and understanding information
   ⚪ Wanting to be a good patient

The majority of participants presented with symptoms to their GP or through emergency hospital admission. Two were diagnosed through cancer screening pathways, and one was referred after an abnormal routine blood test. Over half of the participants had experience of the healthcare system through their own comorbidities, supporting their friends or relatives, or because they had worked in it, but for others it was entirely new. Most reported developing familiarity with the system, processes, and language over time.

### Experiences and decision making

#### Supportive staff in an imperfect system

Participants valued the care shown by individuals within the healthcare system but were aware of its weaknesses. Standardised processes could seem impersonal, particularly during the diagnostic pathway, making an often-unfamiliar experience even more challenging. In the early stages it wasn’t always easy to know who to contact, and the emotional support some needed was not readily available, creating additional anxiety:

*When we were sent away from the hospital, we were given nothing in terms of support, or how to get support*. *All the support that we do have, we’ve had to get, find ourselves*. *I mean in the first, few weeks, after coming out of there, I was throwing out rubber rings everywhere, trying to find someone to talk to (Participant 002, 61–70, male, NHLD)*

Doubt crept in when participants perceived failures in their care, were given conflicting information or plans changed. This caused them to take a step back and process this new information before moving forwards.

Despite these issues, most reported a good experience of care. Healthcare teams were highly regarded and perceived to be supportive, and whilst not always the case at the beginning, once established under a team, participants knew there was someone they could call with questions, and those who had done so found this a positive experience.

#### Additional pressure from COVID-19

Many described an awareness of the additional pressure the NHS was under due to the COVID-19 pandemic, and this affected the care they received. One participant with stable disease felt he had been set aside after two telephone consultations he was expecting didn’t happen. Others felt they missed out because of reduced face-to-face appointments, making it harder for some to seek information:

*I think when you go face to face* *, you seem to be able to come out with more questions… which you should be able to do on a video, but… perhaps it’s just a mental thing talking to somebody face to face, sort of in person (Participant 013, 71–80, male, HLD)*

When participants did attend hospital, there was less opportunity for family to be present, and several reported being alone when receiving their diagnosis.

The restrictions to social contact, risk of catching COVID during anticancer treatment and vaccine side effects placed an additional burden on participants.

#### In the expert’s hands

Participants described putting their trust in those looking after them to act in their best interests. There was a general feeling the decision regarding treatment had already been made by the doctor or wider team, and most described going along with this recommendation. Some took comfort knowing set care pathways were being followed, and a team approach was taken to determining care. Several also felt treatments wouldn’t be offered if not worthwhile or cost effective:

*I’m one of the sorts of people that, when I look at something like the oncologist, he knows a hell of a lot more than I do*. *So, if he’s recommending, then I’ll go with it… because he wouldn’t be recommending if he didn’t think it would do me any good (Participant 040, 71–80, male, HLD)*

When asked whether she had been involved, one participant didn’t feel it was her place to decide on treatment, and this was best left to healthcare professionals. She assumed each time a change was suggested it was to try something ‘better’, i.e. a treatment which was more effective at shrinking the cancer, when often the efficacy of anticancer treatment is lower in later lines of therapy.

Turning down a recommendation in favour of ‘doing nothing’ was seen as foolish, or giving in to death. Whether participants described themselves as ‘tough’, a ‘fighter’, or as someone who goes along with things and does as they’re told, all ultimately accepted treatment.

*I know possibly you could just turn round and say I don’t want that… but*, *faced with what you’re faced with, to me, that would be rather silly (Participant 012, 61–70, female, HLD)*

The few who considered declining treatment felt they were too old, had been through too much, or were feeling low emotionally, but eventually decided to go ahead. One was persuaded by his family, another by a friend and thoughts of family, a third was convinced after hearing a story of treatment success, and the last went along with the recommendation made by their consultant.

Consent was considered a necessity to cover those delivering treatment and an expected step in agreeing to a plan. One participant, however, described having a ‘get out clause’ by not reading the information he had been given, almost abdicating responsibility for his part in the decision to commence treatment.

*Yeah I think it was given to me in a way I understand but what I would say is… all the written information that I was given, I didn’t always read it… So there’s a kind of get out clause there… But it could be that the hospital did say, here’s your information about radiotherapy, if you look on the page three, this tells you about symptoms and side effects, what you might find is it’s very difficult to eat… But I don’t remember anybody kind of sitting with me and telling me that (Participant 021* *, 51–60, male HLD)*

#### Treatment not so bad

Participants had undergone various treatments, and some had received multiple lines of systemic anti-cancer therapies. Some described pre-formed ideas of treatments from the media or prior reading, and were surprised and grateful when their own experiences were better. However, most reported side effects, in some cases life threatening. Several attended hospital or had to stop because of toxicity, but this was generally downplayed, and treatment was perceived to be tolerable:

*On my first week of having it, I ended up getting septicaemia*. *I was rushed in… And they didn’t think I was going to make it… I swam through the chemo, it was the radiotherapy that caused me the trouble. Chemotherapy was fine, I was sick once (Participant 027, 51–60, female, HLD)*

Participants appeared prepared to put up with side effects, accepting they were part of the recommended pathway. Evidence of success in the form of physical changes, investigation results and continuing survival were encouraging, as was being in a position to receive treatment, with participants generally hopeful assessments would allow ongoing therapy.

### Challenges

#### Emotional hurdles

Despite many presenting with symptoms, the diagnosis often came out of the blue and for most was a shock. Some described finding it particularly difficult to accept when they felt so well, or had been healthy before. Many found it hard to take in much of what was said after hearing their diagnosis, and one described her reaction when advised a Do Not Attempt Cardiopulmonary Resuscitation (DNACPR) decision would be appropriate:

*You’ve just been told what you’ve got, and then they come out with, I’m going to put non-resuscitation… I said you are! What gives you the right to tell me I can’t be resuscitated? …Well, we feel you wouldn’t benefit from it, I said well, I want to be resuscitated*. *I don’t care if I come back, a vegetable or this or that, I said no, I said I want to come back… that’s so frightening to hear all this! (Participant 027, 51–60, female, HLD)*

The timing of this conversation may have suggested death was even more imminent than previously imagined, creating additional fear. Despite being otherwise happy to follow professionals’ recommendations, the participant was terrified by this conversation, making it even harder to fully comprehend her situation.

Some participants were grateful for the opportunity to spend time with family, which other acute life-threatening conditions may not have allowed. One worried his prognosis might be so short he wouldn’t have time to put his affairs in order, a fear only allayed when he was prescribed a cycle of treatment longer than he had expected to survive.

*Once I knew I was on a course of pills, it suddenly hits you, your brain sparks and you think well, they think I’ve got enough time left on this planet to take all this course of pills*, *it’s not as though it’s, as urgent as I might have thought… Suddenly you think right that’s it, I don’t s’pose I’ll see the outside world again, that’s it I’ll be rushed in for the rest of my life and then put in a hospice… I mean I still don’t know how urgent it is, but all I know is, it doesn’t seem as bad as I thought, you know? (Participant 037, 51–60, male, HLD)*

Uncertainty about the future featured in most participants’ accounts, making it difficult to plan ahead.

#### Accessing information to further understanding

Participants trusted information provided by healthcare professionals. Though most recalled being told some treatment side effects, written information was often given to reinforce this and provide further detail. Several reported being given too much information at once, some of which seemed irrelevant at the time and was put aside. Some didn’t feel the need to read the information, relying on what they had been told, and some found the language too difficult:

*She told me… she was going to give me two types of drugs… which I’ve never heard of and never remember the names of*. *And then she gave me two very technical sheets on those… which quite honestly was complete gibberish to me (Participant 001, 71–80, male, NHLD)*

Many described only being able to take in a proportion of the information they were given, having to pick out the details they felt were important. Some felt able to process more later after things had had a chance to sink it, but this was often after key information had been imparted and decisions made, and such conversations were not always revisited. Others didn’t get the specific information they wanted. One specifically asked about prognosis but did not get a satisfactory answer, and an opportunity to improve her understanding was missed. Several accessed their records, sometimes looking up medical words, and one suggested a summary document might help them keep track of changes to their disease.

Many searched for additional information online, though noted the internet was not always a reliable source. Some participants sought others’ experiences through support groups or online forums. Many relied on support from friends and family to navigate the system, seek and understand information, and make decisions.

#### Wanting to be a good patient

Despite most participants reporting being able to speak up and ask questions, many described a hesitancy to do so. Some did not want to be a burden by bothering busy healthcare professionals. Others worried their questions would be considered trivial:

*Sometimes I’m a little bit hesitant to phone up asking about those sort of things? …* *maybe, I feel, am I wasting somebody’s time, just asking that? But it is important to me? (Participant 023, 51–60, female, NHLD)*

Most were mindful clinicians were busy and didn’t want to take valuable time away from other patients. They instead relied on planned contacts, which could be a ‘lifeline’, especially when not receiving treatment. One described a reluctance stemming from childhood when he was chastised by a doctor for wasting his time. Others were concerned their team might think badly of them. The one participant who initially declined treatment believed the relationship with her consultant was damaged after she ‘refused’ treatment. She ‘begrudgingly’ gave consent without fully understanding the possible side effects, and later regretted the decision.

*I thought, I’ve got this far, I’ve come back to see him, I’m not gonna waste the man’s time*, *just sign it… and, begrudgingly… I know for a fact I wouldn’t have had treatment if I’d known the side effects… I have had none of the symptoms that are mentioned on that piece of paper, but when I read the paper I thought ooh, I wish I hadn’t booked this (Participant 042, 71–80, female, NHLD)*

## Discussion

The experiences of participants in this study highlight challenges for SDM amongst patients with incurable cancer and health literacy difficulties, and areas for improvement. Though some issues are specific to those experiencing HL difficulties, many are relevant to all patients and have wider implications for SDM in incurable cancer. This has been highlighted below where applicable.

Anxiety and distress associated with such a diagnosis may negatively impact individuals’ ability to process information [20, 21] regardless of health literacy, and many participants found it hard to absorb information after being told difficult news. Addressing patients’ emotional needs is therefore key, ensuring they have time and support to process the information given. Patients should be encouraged to contact their teams with further questions, for clarification and support, and it may also be helpful for a member of the team to make contact after the appointment, to establish understanding, re-explore these issues and address outstanding queries, particularly when patients may be reluctant to initiate this themselves. A clear list of available services may make it easier for people to access additional emotional support if wanted. The fundamental role of the CNS as a crucial member of the multidisciplinary team has been demonstrated, through providing both emotional and practical support, and acting as a single point of contact, and this role must be protected and supported.

Although participants generally found the written resources easy to understand, some would have preferred simpler language, and several reported information overload. Ensuring resources are easily available, written in line with HL principles and offered beyond the first contact might ultimately allow people to better process the information. Greater availability of audio-visual recordings may give patients more control over the information they can access, facilitating better understanding for those who don’t find written resources helpful. Having an easily understandable, personalised record may provide clarity for others, allowing them to track changes to their disease and better informing their decisions. Whilst some may ultimately prefer greater detail, ensuring access to easily understandable information may make it easier for patients and families to digest, regardless of health literacy, particularly in the earlier stages when emotional influences can make it all the more difficult to process the given information.

The participants’ experiences suggest an ongoing power imbalance. As the less powerful actors, patients may not have the capacity or ability to mobilise the necessary resources to fully participate in decision making, whatever their level of HL [22]. However, whilst clinicians are experts in diseases and treatments, patients’ priorities should be discussed and used to inform decisions. Highlighting the value of patients’ expertise in themselves and providing permission to actively contribute to decision making may help foster a more balanced partnership, whilst follow up calls may provide additional opportunities for patients to raise concerns they may otherwise have kept quiet.

Participants experienced acute and potentially serious short-term toxicities of treatment, including life threatening infections, as well as more chronic side effects with a significant impact on day to day life, such as fatigue. Despite this, treatment was generally considered tolerable, perhaps because such toxicities were expected by patients and therefore accepted. Downplaying or acceptance of these toxicities could influence future decisions about treatment and potentially create conflict. It may also result in the patient unnecessarily experiencing a poorer quality of life if an open discussion of the impact of treatment and potential mitigating strategies, such as dose reductions or delays, has not been had.

Consistent with previous research [23], participants reported following recommendations made by clinicians acting in their best interests. However, individuals with lower HL in the palliative phase of illness may be particularly susceptible to persuasion [24]. There is not always equipoise between two options, and there is often a trade off in quality of life when pursuing life-extending treatments, but the value placed by patients on the possible outcomes may differ. Clinicians must seek the patient’s perspective and counsel them through these difficult conversations, being mindful of presenting full and clear information to support decision making.

It is also important to engage with family, friends, and advocates, and identify those who might benefit from additional social support. Groups or online forums can be a valuable resource for patients to support each other, and signposting to relevant groups may facilitate access.

Whilst remote consultations can allow greater flexibility for patients and staff, the reduced face-to-face contact may make it harder for patients with HL difficulties to further their understanding. Video interviews were not possible for some participants, with others preferring telephone, potentially restricting communication through the loss of non-verbal cues. In keeping with a recent cross-sectional study of telehealth visits during the pandemic [25], those with HL difficulties tended to proceed with telephone rather than video interviews. Patients should be offered a choice when planning appointments, taking account of their individual preferences and capabilities, to ensure they are given the opportunity to fully participate and benefit from their consultations with healthcare professionals.

The study was carried out during the COVID-19 pandemic, and some of the challenges, such as being alone when receiving a diagnosis, lack of face to face contact, and, potentially, increased pressure to have DNACPR conversations earlier than might usually happen, may reflect the additional pressure of the pandemic. However, these issues are important at any time and remain relevant to current clinical practice.

Health literacy was assessed using a validated screening tool, however, issues limiting the tool’s ability to distinguish between those with and without HL difficulties were encountered. Answers did not always align with participants’ responses throughout the interview, and some appeared to misinterpret the questions. As in the tool’s validation study [16] the questions were delivered verbally to avoid participants having to read, but some may have misheard. Other participants struggled to choose an answer. They were supported to make a selection, but when this was not achieved, a judgement was made based on the response given. Whilst it is useful to be able to efficiently categorise HL through standardised questioning or assessment, we did not feel the tool correctly captured participants’ HL in this study, and further qualitative examination of such measures is needed to ensure they are truly reflective of individuals’ HL according to more recent and comprehensive definitions.

### Study limitations

Due to the recruitment and consent processes, it was not possible to confirm participants’ medical history until after the interview. This led to the erroneous recruitment of one individual receiving adjuvant treatment. As specific decisions are quite different, this participant’s responses were not included in the main analysis, though elements relevant to general aspects of care were considered. Additionally, further demographic details including ethnicity, education and socioeconomic status were not collected, but may also have influenced participants’ experiences of decision making.

### Clinical implications

Many of these recommendations can be incorporated into routine clinical practice to improve patient experience. Providing information in lay language, available in a range of formats to suit individuals’ preferences, and simplifying processes, will make navigating this complex environment easier for all patients. Ensuring patients are well supported from the outset, enabling them to take in and process information relating to their diagnosis and available options, is also vital.

Findings from this study will be used to design an intervention aimed at supporting shared decision making in the setting of incurable cancer, addressing the specific challenges faced by those experiencing health literacy difficulties to further improve patient care.

## Conclusion

These findings demonstrate the challenges faced by patients with assumed HL difficulties when making decisions about care for incurable cancer. The significant emotional burden that accompanies a diagnosis of incurable cancer may limit the information patients can process, leaving them with an incomplete understanding of their disease and available options. Power imbalance can make it hard to turn down an expert’s recommendation and the patient’s expertise may go unrecognised. Providing joined up support through good teamworking is important, and allowing flexibility in care delivery at an organisational level will foster an environment more conducive for decision making to take place. To achieve success in supporting SDM in this setting, it will be important to consider these challenges and address the combination of psychological, social and informational needs.

## Supporting information

S1 File Interview topic guide. (DOCX)
