# Supplementary material for: The experiences and decision making of patients with incurable cancer and health literacy difficulties
Source: PLoS One. 2024 Oct 3;19(10):e0309104. doi: 10.1371/journal.pone.0309104 (PMC11449316; doi:10.1371/journal.pone.0309104)
Supplement: S1 File — (DOCX) [file pone.0309104.s001.docx]

Interview topic guide

Thank you for agreeing to take part in this interview today. Please answer questions freely and let me know if you want to stop at any point. I will be recording the interview.

I will start the recording now

**<start recording>**

We know that people diagnosed with cancer are often given lots of complicated information and are sometimes faced with some very difficult decisions. We want to find out about how people are given information about their diagnosis and treatment, and whether it is easy to understand. We want to know if there are things that are particularly difficult to understand, so that we can look at how to make them clearer. We also want to find out how we can help patients to speak up and be more involved in decisions about their care, if they want to.

Our goal is to make sure that we give people the information they need, in a way that they can understand, so that they can be involved in decisions about their health.

(Background)

Before we start talking a bit more about that, I’d like to find out a bit about your background if that’s OK?

Would you mind telling me a bit about yourself, like how old you are, whether you are currently working and what sort of work you do?

And if you feel able to, please could you tell me a little bit about your cancer and what treatments you have had? Don’t worry at all if you don’t know all the details.

How much of the day are you able to be up and about? What sort of things are you able/not able to do? (Assessment of ECOG performance status)

(Questions relating more to functional health literacy - refers to the ability to understand health information to improve knowledge and be able to navigate the health system)

1. I’d like you to try and think back to a time when you started a new treatment for your cancer. This may have been surgery, chemotherapy, radiotherapy or something else. Can you tell me a bit about that experience?
   1. How were you given information about this treatment?
   2. Was the information given to you in a way that you could understand?
   3. Did you feel that you completely understood the benefits and risks or side effects of the treatment so that you could make up your own mind about it?
   4. What were you told about any alternatives to this treatment?
   5. Did you try to find out any more about the treatment or alternatives other than what your doctor or nurse told or gave you?
      1. How did you try to do this?
   6. Did you have to sign a consent form for treatment?
      1. How did you feel about doing this?
   7. How did you feel about this whole process of deciding whether to have this treatment?
2. We know that there can be a lot to get your head around when diagnosed with cancer. Especially when people use medical words, it can feel like there is a whole new language to learn.

Lots of people tell us that they sometimes need help understanding this new medical information or filling out forms. We think that more could be done to help make it easier for people understand.

- 1. What are the types of information or forms that you have found or think might be the most difficult to understand?
  2. Has it ever been difficult to know where to go for your appointments, how to get there or what they are for?
     1. Can you tell me a bit about what happened?
  3. How do you deal with things like this when they are difficult?
  4. When things like this aren’t easy to understand, how does that make you feel?

(Questions relating more to critical health literacy - refers to the critical appraisal of information and ability to engage in shared decision making)

1. Have you ever found it difficult to make a decision about your care or treatment because you didn’t feel you had enough information or things hadn’t been explained clearly?
   1. Could you tell me more about this?
   2. Do you feel that you understand enough about your cancer and how it affects your health to be able to make these decisions?
   3. How much have you felt able to ask questions about your care or treatment?
      1. Please could you tell me more about this?
   4. How confident have you felt telling your doctor or nurse what is important to you when thinking about treatment decisions?
   5. Do you feel that you have been involved in decisions about your care?
      1. Can you tell me how?
   6. Have you been as involved in decisions about your care or treatment as you would like?
      1. If not, then can you tell me why you think this is?

(Questions relating more to interactive health literacy - refers to an individual’s ability to act independently on this information, with increased self-confidence and successful interaction with healthcare services)

1. We sometimes have to make decisions about what to do between hospital appointments if we feel unwell or aren’t sure about something. How do you go about this?
   1. Do you feel able to contact your medical team if you have any worries or questions?
   2. How would you do this?

(Chew questions)

1. I’d now like to ask you three short questions about how easy or hard you find it to be involved in your care
   1. How often do you have someone (like a family member, friend, hospital/clinic worker or caregiver) help you read hospital materials?

*None of the time, a little of the time, some of the time, most of the time, all of the time*

- 1. How often do you have problems learning about your medical condition because of difficulty understanding written information?”

*None of the time, a little of the time, some of the time, most of the time, all of the time*

- 1. How confident are you filling out forms by yourself?

*Not at all, a little bit, somewhat, quite a bit, extremely*

1. If you did find it difficult to do things like filling in forms and reading medical information, would you want your doctor or nurse to know?
   1. Why/why not?

(Questions about how to help support)

1. What more do you think could be done to help make it easier for people to receive information about their cancer and treatments?
   1. Do you have any ideas about how things could be done differently?
   2. Would it have helped if you had been given information in a different way or the forms were made easier to understand?
      1. What could have been different?
   3. Some people find that they can understand and remember things better if they see them in pictures or a video, or can listen back to what was said.
      1. Have you ever been given information in this way?
      2. Do you think this would help? Why?
      3. Would you like to be given information in this way?

Thank you very much for answering my questions.

Is there anything that you would like to ask me, or would you like to make any other comments about the things we have discussed today?

Thank you very much again for your time. I will now stop the recording.

**<stop recording>**

Health literacy is the combination of **personal competencies** and **situational resources** needed for people to **access**, **understand,** **appraise** and **use** **information** and **services** to **make decisions** about health. It includes the capacity **to communicate**, **assert** and **act** upon these decisions
